# Exploring marine toxins: comparative analysis of chemical reactivity properties and potential for drug discovery

**DOI:** 10.3389/fchem.2023.1286804

**Published:** 2023-11-01

**Authors:** Norma Flores-Holguín, Joan S. Salas-Leiva, Erick J. Núñez-Vázquez, Dariel Tovar-Ramírez, Daniel Glossman-Mitnik

**Affiliations:** ^1^ Centro de Investigación en Materiales Avanzados SC, Chihuahua, Mexico; ^2^ Centro de Investigaciones Biológicas del Noroeste, La Paz, Baja California Sur, Mexico

**Keywords:** marine toxins, harmful algal blooms, hydrophilic and lipophilic toxins, drug discovery, computational chemistry, conceptual DFT, chemical reactivity properties, bioavailability scores

## Abstract

Marine toxins, produced by various marine microorganisms, pose significant risks to both marine ecosystems and human health. Understanding their diverse structures and properties is crucial for effective mitigation and exploration of their potential as therapeutic agents. This study presents a comparative analysis of two hydrophilic and two lipophilic marine toxins, examining their reactivity properties and bioavailability scores. By investigating similarities among these structurally diverse toxins, valuable insights into their potential as precursors for novel drug development can be gained. The exploration of lipophilic and hydrophilic properties in drug design is essential due to their distinct implications on drug distribution, elimination, and target interaction. By elucidating shared molecular properties among toxins, this research aims to identify patterns and trends that may guide future drug discovery efforts and contribute to the field of molecular toxinology. The findings from this study have the potential to expand knowledge on toxins, facilitate a deeper understanding of their bioactivities, and unlock new therapeutic possibilities to address unmet biomedical needs. The results showcased similarities among the studied systems, while also highlighting the exceptional attributes of Domoic Acid (DA) in terms of its interaction capabilities and stability.

## 1 Introduction

Marine toxins refer to harmful substances generated by a variety of marine microorganisms, particularly dinoflagellates, diatoms, cyanobacteria and bacteria. These microorganisms can undergo rapid growth and form what is commonly known as Harmful Algal Blooms (HAB), leading to the extensive production of these toxins. Consequently, the marine ecosystem suffers adverse effects. Certain marine organisms, such as bivalve mollusks, gastropods, crustaceans and fishes, consume the algae that produce these toxins. This poses a significant risk to human health if these marine products are consumed, causing catastrophic economic consequences for the fishing industry (Martín et al., 1996; Botana et al., 2012; Janssen, 2019; Rotter et al., 2021; Pradhan et al., 2022).

Marine toxins can be categorized into two main groups: hydrophilic and lipophilic. The primary hydrophilic marine toxins include Domoic acid (DA), Saxitoxins (STX), and Tetrodotoxins (TTX). Among the lipophilic marine toxins, we find Okadaic acid (OA), Ciguatoxins (CTX), Pectenotoxins (PbTXs), Yesotoxins (YTX), Azaspiracid (AZA), Cyclic Imines (CI), and Brevetoxins. Additionally, there is a marine toxin class of the amphipathic type, which exhibits both hydrophilic and lipophilic properties. This category includes Palytoxins (PLTXs) (Daguer et al., 2018; Alves et al., 2019; Louzao et al., 2022).

Marine toxins can give rise to a range of syndromes depending on the specific toxin involved and its concentration. These syndromes primarily manifest as gastroenteric, cardiovascular, and neurological symptoms (Sobel and Painter, 2005; Gerssen et al., 2010; Vilariño et al., 2018). The effects of marine toxins on the human body can vary widely, with each syndrome presenting its distinct set of symptoms and clinical signs. It is important to note that the severity and duration of these syndromes can vary depending on the specific toxin, its concentration, and the individual’s sensitivity or exposure level. Prompt medical attention should be sought if any symptoms related to marine toxin exposure are observed Liu et al. (2019); Wu et al. (2019).

The marine environment harbors an astonishing array of chemical and biological diversity when it comes to toxins. This vast diversity not only presents challenges in understanding and mitigating the risks associated with marine toxins but also offers a very great potential as an extraordinary resource for new drug discovery (Ding et al., 2017; Xie et al., 2017) The unique chemical structures and mechanisms of action exhibited by marine toxins make them interesting candidates for drug development (Pradhan and Ki, 2022). Researchers and scientists have recognized the value of exploring marine toxins as a source of potential therapeutic agents and biotechnological applications (Altmann, 2017; Zhao et al., 2019; Ji et al., 2021). By studying the complex interactions between these toxins and their biological targets, valuable insights can be gained into the underlying molecular mechanisms of diseases and physiological processes (Xie et al., 2017). The study of marine toxins has allowed us to not only understand the etiologies of various syndromes due to the consumption of marine products, but also the effect of other routes of exposure such as respiratory, topical and ophthalmic on public and animal health. The potential therapeutic applications of these marine toxins encompasses a large number of diseases like cancer, Alzheimer, diabetes, pain, AIDS, inflammation and schizophrenia (Ji et al., 2021).

Numerous publications have extensively covered the toxic aspects of these molecular systems, including their distribution, detection, characterization, biosynthesis, mitigation, and other related features. These studies span across disciplines such as toxicity, hazardous materials, marine pollution, environmental contamination, food safety, and various analytical chemistry journals (Nelis et al., 2021; Kong et al., 2023). However, to date, there has been a lack of research that systematically characterizes the molecular properties of toxins and explores similarities or differences among them, despite their diverse structures.

In this study, we conducted a comparative analysis of the reactivity properties of two hydrophilic and two lipophilic marine toxins. These were chosen because Ciguatoxins (CTX) and Saxitoxins (STX) are the most common marine toxin causing illness (Friedman et al., 2017). The names, abbreviations, chemical formulae, and molecular structures of the studied systems are presented in Table 1. The significant differences in their molecular structures and the contrasting definitions of lipophilic and hydrophilic properties make both types of systems intriguing in the field of drug design. Considering the definition of lipophilicity, which refers to a substance’s ability to dissolve in lipids or fats, and its correlation with biological activities, this physical property plays a crucial role in determining the potency of a drug to distribute throughout and eliminate from the body. As the majority of body fluids are hydrophilic, the body employs specific carriers capable of binding with lipophilic substances, facilitating their transportation to the intended targets. In contrast, hydrophilic molecules are predominantly polar, or at least possess a polar region. These molecules exhibit a propensity for forming hydrogen bonds and readily bonding with targets when employed as ligands. This makes them favorable candidates for therapeutic interventions. The exploration of both lipophilic and hydrophilic properties in drug design is essential due to their distinct implications on drug distribution, elimination, and target interaction. By understanding the reactivity properties of these toxins and their relationship to drug design, researchers can gain valuable insights into optimizing drug candidates for enhanced therapeutic outcomes (Parr and Yang, 1989; Geerlings et al., 2003; Clark, 2005; Gázquez et al., 2007; Chattaraj et al., 2009; Tsaioun and Kates, 2011; Wang and Urban, 2014; Kallen, 2019; Geerlings et al., 2020; Glossman-Mitnik, 2022; Liu, 2022; Kaya et al., 2023).

**TABLE 1:** The names, abbreviations, chemical formulae, and molecular structures of the marine toxins considered in this study.

Name	Abbreviation	Chemical formulae	Molecular structure
Saxitoxin	STX	C_10_H_17_N_7_O_4_	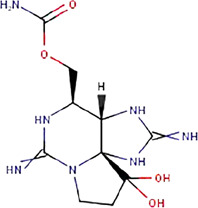
Domoic Acid	DA	C_15_H_21_NO_6_	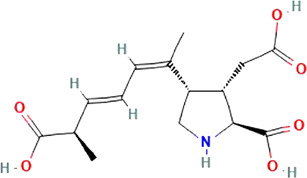
Okadaic Acid	OA	C_44_H_68_O_13_	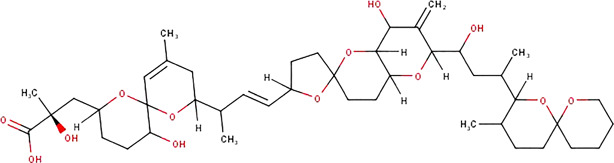
Ciguatoxin	P-CTX-4B	C_60_H_84_O_16_	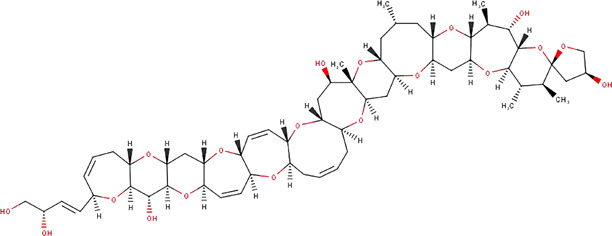

Thus, the aim of this study is to fill this gap by examining the molecular systems of toxins and identifying potential similarities through the analysis of global reactivity and bioactivity properties. By investigating these similarities, we can gain insights into their potential as precursors for the development of new drugs. Through this research, we seek to uncover commonalities in the reactivity profiles of toxins, despite their structural disparities. This comparative analysis will provide valuable information for understanding the underlying mechanisms of toxin activity and exploring their potential as sources of novel therapeutic agents (Pennington et al., 2018).

By elucidating the shared molecular properties among toxins, we can identify patterns and trends that may guide future drug discovery efforts. Additionally, this research has the potential to contribute to the field of molecular toxinology, facilitating a deeper understanding of the relationship between toxin structures and their bioactivities. The findings from this study will contribute to the existing body of knowledge on toxins and provide a foundation for further investigations into their potential applications in drug development. The outcomes of this study enable us to identify the most promising toxin for potential drug applications based on its reactivity, pharmacokinetics, and bioavailability properties. These findings facilitate the exploration of molecular docking between the toxin and a protein receptor within the human body’s system. Ultimately, the goal is to leverage these molecular systems to unlock new therapeutic possibilities and address unmet medical needs.

## 2 Computational methodology

The initial structures were sourced from the PubChem database (https://pubchem.ncbi.nlm.nih.gov/) and examined using the Marvin View17.15 program (ChemAxon, Budapest, Hungary) to identify the most stable conformer utilizing Molecular Mechanics (Halgren, 1996a,b, 1999; Halgren and Nachbar, 1996; Halgren, 1996c). The initial conformers are subjected to relaxation at the molecular mechanics level using the MMFF94 force field, employing a strict optimization limit. A maximum of 100 conformers are generated with a diversity limit of 0.1 kcal/mol. Subsequently, the defined molecular system underwent gas phase optimization. After optimization, vibrational frequencies were calculated to ensure the attainment of true minima. Energy calculations were then conducted to predict the reactivity descriptors based on Conceptual Density Functional Theory (CDFT) (Parr and Yang, 1989; Geerlings et al., 2003; Gázquez et al., 2007; Chattaraj et al., 2009; Geerlings et al., 2020; Glossman-Mitnik, 2022; Liu, 2022; Kaya et al., 2023).

For the optimization process, the semi-empirical PM6 method (Stewart, 2007; Frisch et al., 2016) was employed. In the optimization phase, our research team has devised a computational approach. This method first identifies the most stable conformer and subsequently refines it using the semiempirical PM6 technique. Notably, these geometrical outcomes have demonstrated a high level of reliability comparable to that of traditional quantum mechanical methods. Electronic properties were computed (Frisch et al., 2016) using the Def2TZVP basis set (Weigend and Ahlrichs, 2005; Weigend, 2006) and the MN12SX density functional (Peverati and Truhlar, 2012), with water as the solvent and the SMD solvent model (Marenich et al., 2009). The KID procedure (Flores-Holguín et al., 2019a; Frau et al., 2019; Flores-Holguín et al., 2019b, 2020a,b,c; Flores-Holguín et al., 2021) was applied, incorporating the results obtained from the optimization, frequency calculations, and energy calculation of the ground states to obtain geometries and electronic properties (Young, 2001; Frisch et al., 2016).

Once the CDFT calculations were obtained, the systems underwent prediction of bioavailability scores. Additionally, a compact depiction of the characteristics of the molecules related to their bioavailability can be displayed in a graphical mode through the so-called bioavailability radars (Clark, 2005; Tsaioun and Kates, 2011; Wang and Urban, 2014; Kallen, 2019).

### 2.1 Theoretical background

#### 2.1.1 CDFT and chemical reactivity

Conceptual Density Functional Theory (DFT) serves as a powerful tool for chemists, providing them with a comprehensive framework consisting of well-defined chemical concepts. This framework not only facilitates a qualitative understanding but also enables the quantitative prediction of chemical reactivity. When a molecule engages in a reaction, fundamental changes occur in its electron population. These changes manifest as an increase in the number of electrons during nucleophilic attack or a decrease during electrophilic attack (Parr and Yang, 1989; Geerlings et al., 2003; Gázquez et al., 2007; Chattaraj et al., 2009; Geerlings et al., 2020; Glossman-Mitnik, 2022; Liu, 2022; Kaya et al., 2023).

Moreover, the external potential experienced by the electrons within the molecule undergoes a transformation. Previously, the electrons were solely attracted to the nuclei of the molecule. However, with the introduction of an attacking reagent, the electrons now experience the combined influence of being attracted to the nuclei while also being repelled by the electrons of the attacking species. As a result, the susceptibility of a molecule to chemical reactions is determined by its response to two primary factors: a) alterations in the number of electrons and b) modifications in the external potential (Parr and Yang, 1989; Geerlings et al., 2003; Gázquez et al., 2007; Chattaraj et al., 2009; Geerlings et al., 2020; Glossman-Mitnik, 2022; Liu, 2022; Kaya et al., 2023).

By analyzing these two aspects, Conceptual DFT unveils crucial insights into the reactivity of molecules. It allows chemists to comprehend the intricate interplay between electron dynamics and external influences, paving the way for a comprehensive understanding of chemical transformations. Through the application of Conceptual DFT, chemists can predict and elucidate key aspects of chemical reactivity, thereby empowering them to design and optimize chemical reactions with greater precision. The ability to assess a molecule’s response to changes in electron population and external potential fosters the development of innovative strategies for molecular manipulation and synthesis, bolstering advancements in various fields such as drug discovery, materials science, and catalysis (Parr and Yang, 1989; Geerlings et al., 2003; Gázquez et al., 2007; Chattaraj et al., 2009; Geerlings et al., 2020; Glossman-Mitnik, 2022; Liu, 2022; Kaya et al., 2023).

It has been shown that considering functional differentiability of the electronic energy E with respect to N and ν(r), a series of response functions emerge, corresponding to a hierarchy of different levels. At the foundational level of this hierarchy, the response functions capture the system’s overall sensitivity to variations in the electron count N. This knowledge sheds light on the fundamental relationship between electron population and the resulting electronic energy, forming the basis for understanding the system’s behavior as electrons are added or removed. Indeed, in the first level the most famous and considered response function is the chemical potential μ = (*∂E*/*∂N*)_ν(*r*)_, which has been established as the negative of the electronegativity χ. In this way, this first CDFT descriptor can be written in terms of the ionization energy and the electron affinity, or in turn, in terms of the Frontier orbitals HOMO and LUMO as: $\chi =-\frac{1}{2}\left(I+A\right)\approx \frac{1}{2}\left(\epsilon_{L}+\epsilon_{H}\right)$ , where ϵ_*L*_ and ϵ_*H*_ are the corresponding energies of the LUMO and HOMO orbitals (Parr and Yang, 1989; Geerlings et al., 2003; Gázquez et al., 2007; Chattaraj et al., 2009; Geerlings et al., 2020; Glossman-Mitnik, 2022; Liu, 2022; Kaya et al., 2023).

The next important CDFT descriptor in the hierarchy is the global hardness η which is equal to ${\left(\partial E^{2}/\partial N^{2}\right)}_{\nu \left(r\right)}$ and can be written for computational purposes as η = (*I* − *A*) ≈ (ϵ_*L*_ − ϵ_*H*_). While the chemical potential μ measures of the tendency of the electron to escape from systems in equilibrium, the global hardness η is an indication of the resistance of the electron cloud to be deformed resulting in a lower reactivity (Parr and Yang, 1989; Geerlings et al., 2003; Gázquez et al., 2007; Chattaraj et al., 2009; Geerlings et al., 2020; Glossman-Mitnik, 2022; Liu, 2022; Kaya et al., 2023).

Several years later, a relationship between χ and η was theoretically derived giving rise to the global electrophilicity ω as $\mu^{2}/2\eta =\left[{\left(I+A\right)}^{2}/4\left(I-A\right)\right]\approx \left[{\left(\epsilon_{L}+\epsilon_{H}\right)}^{2}/4\left(\epsilon_{L}-\epsilon_{H}\right)\right]$ , thus denoting the tendency of a molecule to accept electrons and undergo chemical reactions (Parr et al., 1999).

On the basis of the previous developments, Gázquez, Cedillo and Vela designed theoretically two additional CDFT descriptors: the electrodonating power, or the capability of a chemical system to donate a small fractional charge, as *ω* ^−^ = ${\left(3I+A\right)}^{2}/16\left(I-A\right)\approx {\left(3\epsilon_{H}+\epsilon_{L}\right)}^{2}/16\eta$ , and the electroaccepting power, or the capability of a chemical system to accept a small fractional charge as *ω* ^+^ = ${\left(I+3\;A\right)}^{2}/16\left(I-A\right)\approx {\left(\epsilon_{H}+3\epsilon_{L}\right)}^{2}/16\eta$ , while Chattaraj devised the net electrophilicity as Δω^±^ = ω^+^ − (−ω^−^) = ω^+^ + ω^−^, as a measure of the relative electrophilicity (Gázquez et al., 2007; Chattaraj et al., 2009).

#### 2.1.2 Drug-likeness and pharmacokinetic properties

Drug likeness refers to the intricate balance of diverse molecular properties and structural characteristics that determine the similarity of a given molecule to known drugs. These properties, such as hydrophobicity, electronic distribution, hydrogen bonding traits, molecule size and flexibility, as well as the presence of various pharmacophoric features, play a significant role in influencing the behavior of the molecule within a living organism. They affect essential factors like bioavailability, transport properties, protein affinity, reactivity, toxicity, metabolic stability, and more. One tool that can aid in this analysis is Molinspiration, a Java-based property calculation toolkit available at http://www.molinspiration.com. By utilizing Molinspiration, a set of molecular descriptors can be generated, facilitating the identification of structures lacking drug-like properties and the selection of potential drug candidates. Some of these descriptors are (Clark, 2005; Tsaioun and Kates, 2011; Wang and Urban, 2014; Kallen, 2019):

• LogP is a measure of the solubility of a chemical compound in both water and oil, which is important for understanding how the compound will behave in biological systems. Chemical compounds can be classified as either hydrophilic (water-loving) or hydrophobic (water-repelling). Compounds that are hydrophilic are soluble in water, while those that are hydrophobic are soluble in oil. LogP is a way to quantify how much more soluble a compound is in oil compared to water. LogP is calculated by taking the logarithm of the partition coefficient, which is the ratio of the concentration of the compound in oil *versus* water. The higher the logP value, the more hydrophobic the compound is and the more likely it is to accumulate in fatty tissues in the body. LogP is an important parameter in drug discovery, as compounds with certain logP values may have better efficacy, pharmacokinetic properties, and toxicity profiles. It can also be used to predict how well a drug will penetrate biological barriers, such as the blood-brain barrier, which can impact its effectiveness in treating certain diseases (Clark, 2005; Tsaioun and Kates, 2011; Wang and Urban, 2014; Kallen, 2019).
• TPSA stands for Topological Polar Surface Area, and it is a measure of the polarity of a chemical compound. More specifically, TPSA quantifies the surface area of a compound that is polar or capable of hydrogen bonding Ertl et al. (2000). Polar molecules have partial charges due to differences in electronegativity between atoms, and they can interact with other polar molecules through dipole-dipole interactions or hydrogen bonding. By measuring the TPSA of a compound, researchers can predict its ability to interact with biological systems, such as enzymes or cell membranes. Compounds with a higher TPSA value are more likely to be soluble in water and interact with polar biological molecules, while those with a lower TPSA value are more likely to be hydrophobic and interact with non-polar biological molecules. TPSA is an important parameter in drug discovery, as it can be used to predict the pharmacokinetics and pharmacodynamics of drugs in the body. Compounds with a TPSA that falls within a specific range are more likely to have optimal pharmacokinetic properties, such as good absorption, distribution, metabolism, and excretion, and thus have a better chance of being developed into successful drugs (Clark, 2005; Tsaioun and Kates, 2011; Wang and Urban, 2014; Kallen, 2019).
• Molecular volume plays a crucial role in understanding and predicting pharmacokinetics, which is the study of how a drug moves within the body. The molecular volume refers to the space occupied by a molecule in three-dimensional space. It is a fundamental property that directly affects various aspects of drug behavior within the body. For instance, during the absorption phase, a drug with a larger molecular volume may encounter challenges crossing cellular membranes compared to smaller molecules. This can affect the drug’s bioavailability and overall efficacy (Clark, 2005; Tsaioun and Kates, 2011; Wang and Urban, 2014; Kallen, 2019).
• The Rule of 5, also known as Lipinski’s Rule of 5, is a widely used guideline in drug discovery and medicinal chemistry (Lipinski et al., 2001). It was developed by Christopher A. Lipinski to help identify molecules with a higher likelihood of good oral bioavailability and permeability. The Rule of 5 focuses on four key physicochemical properties of a drug candidate: molecular weight, lipophilicity (expressed as logP or logD), hydrogen bond donors, and hydrogen bond acceptors. According to the rule, for a drug molecule to have favorable oral bioavailability, it should meet the following criteria:
  •Molecular Weight: The molecular weight of the drug candidate should be less than or equal to 500 Da. This criterion helps ensure that the molecule is small enough to be efficiently absorbed through biological membranes.
  •Lipophilicity: The calculated logarithm of the partition coefficient (logP) should be less than or equal to 5. This property measures the molecule’s hydrophobicity or its tendency to dissolve in lipid-based environments. A moderate lipophilicity is desired to strike a balance between solubility in water and permeability through lipid membranes.
  •Hydrogen Bond Donors: The number of hydrogen bond donors (usually represented by the count of -OH and -NH groups) should be less than or equal to 5. Limiting the number of hydrogen bond donors helps ensure that the molecule doesn’t exhibit excessive polarity, which can hinder its ability to cross biological barriers.
  •Hydrogen Bond Acceptors: The number of hydrogen bond acceptors (typically represented by the count of -N and -O atoms) should be less than or equal to 10. Controlling the number of hydrogen bond acceptors helps minimize the molecule’s affinity for water, increasing its likelihood of efficient absorption.

The Rule of 5 is a valuable tool for early-stage drug discovery as it helps filter out drug candidates that may face challenges in terms of absorption and permeation. However, it is important to note that the Rule of 5 is a guideline rather than an absolute rule, and exceptions can be made based on specific cases and additional factors. By applying the Rule of 5 during the drug discovery process, researchers can prioritize molecules that are more likely to possess favorable pharmacokinetic properties. This aids in the development of potential drug candidates with improved chances of reaching their target sites and demonstrating therapeutic efficacy when administered orally (Clark, 2005; Tsaioun and Kates, 2011; Wang and Urban, 2014; Kallen, 2019).

• The concept of the number of rotatable bonds is an important factor to consider in the field of pharmacokinetics. It refers to the count of single bonds that can rotate freely around their axis within a molecule. The presence and quantity of rotatable bonds can significantly influence various aspects of a drug’s pharmacokinetic behavior. The number of rotatable bonds affects drug absorption, distribution, metabolism, and excretion. During the absorption process, molecules with a higher number of rotatable bonds may face challenges in crossing biological membranes efficiently. The increased flexibility of these bonds can lead to larger molecular volumes, making it more difficult for the drug to traverse cellular barriers, such as the gastrointestinal epithelium or the blood-brain barrier (Clark, 2005; Tsaioun and Kates, 2011; Wang and Urban, 2014; Kallen, 2019).

## 3 Results and discussion

**FIGURE 1:**
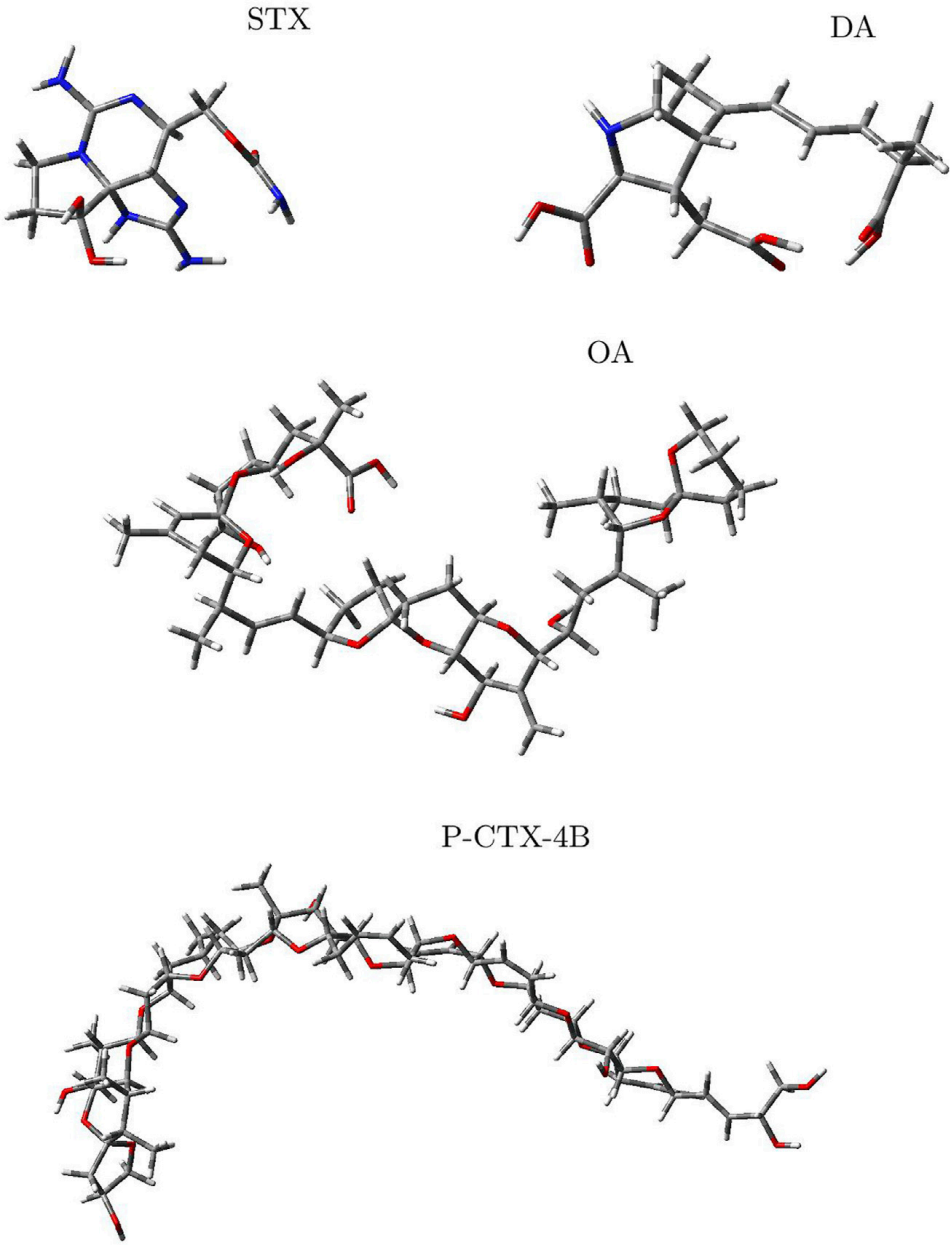
Graphical display of the optimized molecular structures of Saxitoxin (STX), Domoic Acid (DA), Okadaic Acid (OA) and Ciguatoxin (P-CTX-4B).

The molecular structures of the four marine toxins considered in this study were optimized following the computational methodology presented in Section 2, and the results are displayed in Figure 1. As usual, a frequency analysis was done in each case to check for the absence of imaginary frequencies as a mean of verification that the structures corresponded to a minimum on the energy landscape.

The geometries exhibited by the systems under study vary significantly, presenting a wide array of structural arrangements. Consequently, identifying a suitable docking geometry with a defined target becomes a challenging task. Despite the complexity in achieving a precise alignment, the drug-likeness of these systems can be determined by analyzing their reactivity and pharmacokinetics properties.

In addition to their intricate geometries, these systems showcase diverse reactivity profiles that contribute to their drug-likeness evaluation. Understanding how the systems interact with their molecular targets, such as enzymes or receptors, is crucial in assessing their potential as pharmaceutical agents. Reactivity studies shed light on the chemical transformations and interactions that occur between the systems and their targets, providing insights into the systems’ efficacy and specificity.

Furthermore, an evaluation of the pharmacokinetics properties is essential in assessing the drug-likeness of the studied systems. Pharmacokinetics encompasses the processes of absorption, distribution, metabolism, and excretion (ADME) of a drug within an organism. By examining how these systems are absorbed, distributed, metabolized, and eliminated, we gain valuable information about their potential as viable drug candidates. Factors such as bioavailability, half-life, and clearance rate contribute to determining the pharmacokinetic profile and overall drug-likeness.

Despite the challenges posed by the diverse geometries of the systems, a comprehensive analysis of their reactivity and pharmacokinetics properties allows us to assess their suitability as drug candidates. By examining their interactions with targets and understanding how they navigate the complex processes of ADME, we can gain insights into their potential as effective and safe therapeutic agents.

### 3.1 CDFT chemical reactivity

After achieving the ground-state geometry for each molecule, the total and orbital energies were calculated as mentioned in Section 2. The analysis of these results were used to calculate the corresponding CDFT descriptors presented in Section 2.1.1. Besides these descriptors, we believe that it is important to determinate another two additional ones, namely the global softness *S*, defined as the inverse of the global hardness η, and the nucleophilicity index *N* proposed by Domingo et al. (Domingo et al., 2008; Jaramillo et al., 2008; Domingo and Sáez, 2009; Domingo and Perez, 2011; Domingo et al., 2016). This last descriptor was simply defined as *N*_(*Nu*)_ = E_*HOMO(Nu)*_ - E_*HOMO(TCE)*_, where Nu is the molecule of interest and TCE refers to tetracyanoethylene taken as a reference.

The results for the calculation of all these CDFT descriptors for the four marine toxins considered in this study are shown in Table 2:

**TABLE 2:** Global reactivity descriptors: Electronegativity *χ*), Hardness *η*), Electrophilicity *ω*) (all in eV), Softness S (in eV^−1^), Nucleophilicity N, Electrodonating Power (*ω* ^−^), Electroaccepting Power (*ω* ^+^) and Net Electrophilicity (Δ*ω* ^±^) (also in eV).

Marine toxin	A	I	**χ**	**η**	**ω**	S	N	***ω*** ^**−**^	***ω*** ^**+**^	**Δ*ω*** ^**±**^
STX	0.32	6.19	3.25	5.87	0.90	0.17	2.61	4.33	0.54	4.89
DA	1.26	5.81	3.54	4.55	1.38	0.22	2.98	4.81	1.27	6.08
OA	0.61	6.77	3.69	6.16	1.11	0.16	2.03	4.44	0.75	5.19
P-CTX-4B	0.58	6.45	3.52	5.87	1.05	0.17	2.34	4.23	0.72	4.95

Table 2 provides insightful information regarding the electron affinities (EA) of the systems under consideration. Notably, the lipophilic systems exhibit EA values lower than 1 eV. On the other hand, molecule DA displays an EA of 1.26 eV, while molecule STX has an EA of 0.32 eV. It is important to note that the chemical potential, which is the negative of electronegativity, as mentioned earlier, signifies the tendency of electrons to escape from systems in equilibrium. Examining Table 2, we observe that the analyzed systems exhibit chemical potential values ranging from -3.25 (STX) to -3.69 (OA). This implies that STX and P-CTX-4B have a higher propensity to release their electrons more readily.

Moving on to chemical hardness, we find that OA possesses the highest hardness value, followed by STX and P-CTX-4B. It is interesting to note that despite their structurally distinct nature, these systems exhibit varying degrees of chemical hardness. On the other hand, molecule DA demonstrates the greatest capacity to interact with other molecular systems, as indicated by its hardness value of 4.55 eV. This high capacity for interrelation with a specific target highlights its potential significance in various molecular interactions.

The electrophilicity of the systems is a crucial aspect in their ability to stabilize themselves after being saturated with electrons from the surrounding environment. In this context, molecule DA stands out as the system with the highest electrophilic capability. The bonding saturation within this molecule plays a fundamental role in its stability capacity following electron saturation from the surroundings. By having its bonding sites fully occupied, molecule DA effectively maintains its stability, preventing any potential disruptions arising from electron interactions.

In summary, Table 2 provides valuable insights into the electron affinities, chemical potential, and chemical hardness of the systems. Molecule DA demonstrates higher electron affinity compared to the lipophilic systems, while STX and P-CTX-4B exhibit easier electron release. Among the systems, OA possesses the highest chemical hardness, closely followed by STX and P-CTX-4B. Additionally, molecule DA showcases exceptional electrophilicity, primarily due to the bonding saturation that enables its remarkable stability following electron saturation.

### 3.2 Drug-likeness and pharmacokinetics properties

Bioavailability is a crucial factor in determining the potential of a molecule to become a drug, as discussed in the Properties background section. It represents the measure of the molecule’s likelihood to exhibit drug-like properties. In order to evaluate this, a set of properties was calculated using the Simplified Molecular Input Line Entry Specification (SMILES) notations and the results were compiled in Table 3.

**TABLE 3:** A summary of the druglikeness properties estimated with Molinspiration software.

Marine toxin	logP	TPSA	MolecularVolume	Rule of five(Violations)	Number ofRotatable Bonds
STX	-3.09	185	246.75	2	3
DA	-0.91	124	284.07	0	7
OA	2.87	186	760.88	2	10
P-CTX-4B	3.15	241	1012.78	3	3

One of the properties that aids in assessing bioavailability is the LogP, also known as the octanol/water partition coefficient. This coefficient provides insight into how well the molecule is expected to partition between organic elution solvent (octanol) and water. For an orally administered drug, a LogP value less than 5 is generally considered favorable. Ideally, the LogP value falls within the range of 1.35–1.80, indicating optimal partitioning characteristics.

Upon analyzing the LogP values presented in Table 3, it becomes evident that none of the molecules possess the ideal LogP value for an oral drug within the specified range. However, it is noteworthy that all the calculated LogP values are below 5, suggesting that the molecules still exhibit a certain level of partitioning potential.

While the LogP values may not align perfectly with the ideal range, it is important to consider that bioavailability is a multifaceted concept influenced by various factors beyond just the LogP value. Additional properties and characteristics, such as molecular weight, solubility, and permeability, also contribute to the overall bioavailability of a molecule.

In conclusion, Table 3 provides valuable insights into the calculated properties that contribute to bioavailability. Although the LogP values of the molecules do not fall within the ideal range for an oral drug, it is worth noting that they are all below 5, indicating a certain level of partitioning potential. Assessing bioavailability requires a comprehensive analysis of multiple properties and characteristics to determine the drug-likeness and potential of a molecule.

The topological polar surface area (TPSA) has proven to be a valuable predictor of drug absorption (Palm et al., 1996). PSA refers to the combined surface area occupied by oxygen and nitrogen atoms, as well as hydrogen atoms bonded to these electronegative atoms. It has been observed that molecules with a PSA larger than 140 Å^2^ tend to have poor permeability across cell membranes. In the case of the marine toxins under study, their larger PSAs contribute to their overall poor permeability, with the exception of molecule DA, which has a PSA of 124 Å^2^.

Molecular volume is another crucial factor that governs the transport of drugs from the site of administration to the site of action (Bartzatt, 2005). Hydrophilic toxins generally have a molecular volume below 284, indicating their relatively smaller size and favorable transport properties. However, molecules like OA and P-CTX-4B, due to their large molecular structures, have considerably larger volumes, which may impact their ability to efficiently traverse biological barriers.

The Rule of Five is a set of guidelines used to assess the likelihood of a molecule’s bioavailability. According to this rule, molecules that violate more than one of its parameters may encounter challenges in terms of their bioavailability. Among the studied compounds, only molecule DA adheres to all the parameters of the Rule of Five, implying a higher potential for bioavailability compared to the others.

The number of rotatable bonds in a molecule, which represents the bonds capable of free rotation, plays a significant role in molecular flexibility. It has implications for bioavailability and binding potency. In this case, molecule OA exhibits ten rotatable bonds, indicating a high degree of flexibility, which may result in predicted low oral bioavailability. Molecule DA, on the other hand, has seven rotatable bonds, while STX and P-CTX-4B possess only three. This difference in rigidity and flexibility influences the physical structure of the compounds and can have implications for their chemical reactivity and pharmacological properties.

In summary, the polar surface area, molecular volume, Rule of Five compliance, and number of rotatable bonds contribute to our understanding of the bioavailability and physicochemical characteristics of the studied compounds. These factors shed light on their potential for permeability, transport, and reactivity, providing valuable insights for drug development and optimization.

The effectiveness of a drug relies on its ability to reach the target site with the appropriate concentration and bioactive properties to elicit the desired biological effects (Veber et al., 2002). Pharmacokinetics encompasses the processes of drug absorption, distribution, metabolism, and excretion, which govern the passage of drugs into, through, and out of the body (Clark, 2005; Tsaioun and Kates, 2011; Wang and Urban, 2014; Kallen, 2019). Absorption refers to the movement of a drug from the site of administration to the site of action. Distribution involves the drug’s journey through the bloodstream to various tissues in the body. Metabolism involves the breakdown of the drug, while excretion describes its removal from the body (Clark, 2005; Tsaioun and Kates, 2011; Wang and Urban, 2014; Kallen, 2019). To evaluate the relevant properties of both hydrophilic and lipophilic molecules in this study, SwissADME (Daina et al., 2017), a freely available web tool, was used to assess pharmacokinetics and druglikeness.

In terms of drug likeness, SwissADME (Daina et al., 2017) incorporates an additional set of filters. These filters, encompassing five distinct rules, build upon the previously established Lipinski criteria. These rules encompass a diverse array of property ranges within which a molecule is deemed to possess drug-like qualities. These filters underwent evaluation by prominent pharmaceutical companies, aiming to enhance their proprietary chemical libraries. Here’s a breakdown of the filters:

• Pfizer’s Lipinski filter predicts that heightened chances of poor absorption or permeation exist when there are over 5 hydrogen bond donors, more than 10 hydrogen bond acceptors, a molecular weight surpassing 500, and a calculated Log P exceeding 5.
• Amgen’s Ghose filter considers a molecular weight ranging from 160 to 480, molar refractivity within 40–130, and a total atom count between 20 and 70 (Ghose et al., 1998).
• GSK’s Veber filter focuses on 10 or fewer rotatable bonds and a polar surface area equal to or less than 140 Å^2^ (Veber et al., 2002).
• Pharmacia’s Egan filter sets thresholds of Log P less than or equal to 5.88 and polar surface area equal to or less than 131.6 Å^2^ (Egan et al., 2000).
• Bayer’s Muegge filter encompasses molecular weights from 200 to 600, Log P between -2 and 5, polar surface area of 150 or less, no more than seven rings, a majority of carbons over 4, less than 15 rotatable bonds, and fewer than 10 hydrogen bond acceptors and donors (Muegge et al., 2001).

Additionally, the Abbot Bioavailability Score (AAS) assesses the likelihood of a compound having at least 10% oral bioavailability in rats or measurable Caco-2 permeability Martin (2005). This score relies on total charge and polar surface area values, as well as adherence to the Lipinski filter. The scores categorize compounds into four probability ranges: 11%, 17%, 56%, or 85%. A comparison of the drug-likeness predicted for the different molecules by considering the violations to the criteria mentioned in the Computational Methodology section, together with the Abbott Availability Scores (AAS) are displayed in Table 4:

**TABLE 4:** Predicted drug-likeness and Abbott Availability Scores (AAS) for the studied marine toxins.

Molecular system	Lipinski	Ghose	Veber	Egan	Muegge	AAS
STX	2	1	1	1	3	0.17
DA	0	0	0	0	0	0.56
OA	2	3	1	1	3	0.11
P-CTX-4B	3	3	1	1	5	0.17

The findings presented in Table 4 serve to reinforce the earlier observations, underscoring the considerable potential of DA as a noteworthy candidate for an essential therapeutic drug. These results not only corroborate the initial insights but also provide additional substantial evidence to substantiate the notion that DA holds significant promise in the realm of therapeutic applications. However, there have been reported some risks related to DA that must be not overlooked (Saeed et al., 2017; Funk et al., 2014; Grattan et al., 2018).

In order to explore the pharmacokinetics of the molecular systems under investigation, it was imperative to acquire the Simplified Molecular Input Line Entry Specification (SMILES) notations for each individual system. These notations were then utilized in conjunction with the freely available Swiss Target Prediction software, as mentioned earlier, to identify and analyze the associated ADME (Absorption, Distribution, Metabolism, and Excretion) properties. The results of this analysis have been compiled and presented in Table 5.

**TABLE 5:** Absorption, Distribution, Metabolism, and Excretion (ADME) parameters related to the marine toxins considered in this study.

Property	STX	DA	OA	P-CTX-4B
GI absorption	Low	High	Low	Low
BBB permeant	No	No	No	No
P-gp substrate	No	No	Yes	Yes
CYP1A2 inhibitor	No	No	No	No
CYP2C19 inhibitor	No	No	No	No
CYP2C9 inhibitor	No	No	No	No
CYP2D6 inhibitor	No	No	No	No
CYP3A4 inhibitor	No	No	No	No
Log Kp (skin permeation) (cm/s)	-11.41	-9.09	-8.79	–1.34

The ADME properties obtained through this process offer valuable insights into how the studied molecular systems interact with the body and their potential for use as pharmaceutical agents. The Absorption property provides information on the extent and rate at which a molecule is absorbed into the bloodstream after administration. Distribution refers to the process of how the molecule is dispersed and transported throughout the body. Metabolism explores the chemical transformations that the molecule undergoes in the body, while Excretion examines the elimination of the molecule or its metabolites from the body (Buxton and Benet, 2017).

By utilizing the SMILES notations and leveraging the Swiss Target Prediction software, a comprehensive evaluation of the ADME properties has been conducted for the studied systems. This analysis facilitates a better understanding of how these systems may behave within a biological context, including their potential for absorption, distribution, metabolism, and excretion.

The information presented in Table 5 serves as a valuable resource in assessing the pharmacokinetics of the studied molecular systems. It provides an overview of their predicted ADME properties, enabling researchers and scientists to make informed decisions regarding their suitability for further development and potential therapeutic applications.

In summary, the acquisition of SMILES notations and subsequent analysis using the Swiss Target Prediction software has allowed for the determination of the ADME properties of the studied molecular systems. The results presented in Table 5 shed light on their potential behavior within the body, aiding in the assessment of their pharmacokinetic profiles and potential as viable drug candidates.

Table 5 provides insights into the pharmacokinetics properties of the studied systems. It becomes evident that the majority of the systems exhibit low gastrointestinal absorption (GI), with the exception of molecule DA, which displays high GI absorption. This indicates that DA has a higher likelihood of being effectively absorbed through the gastrointestinal tract, potentially enhancing its bioavailability.

Furthermore, none of the studied systems demonstrate blood-brain barrier permeation. This suggests that these systems have limited ability to cross the blood-brain barrier, which can be advantageous or disadvantageous depending on the intended therapeutic application. However, molecule OA stands out as one of the two systems that are substrate of the permeability glycoprotein, an important protein transporter involved in the disposition of many drugs. This indicates that OA may interact with this transporter, potentially influencing its distribution and elimination within the body.

Interestingly, none of the studied systems show interaction with the isoenzyme superfamily of cytochromes P450 (CYP), which are essential factors in drug elimination through metabolic biotransformation. This suggests that these systems may not undergo significant metabolism mediated by CYP enzymes, potentially affecting their clearance rate and overall pharmacokinetic profile.

In terms of skin permeation, all the toxins exhibit high values, ranging from -8.79 to -11.41 cm/s. This indicates that these systems have a significant ability to permeate the skin, which can be relevant in the context of dermal exposure or transdermal drug delivery.

In summary, Table 5 provides important information about the pharmacokinetics properties of the studied systems. The data highlights the differences in gastrointestinal absorption, blood-brain barrier permeation, interaction with transporters and enzymes, and skin permeation. Understanding these properties contributes to the characterization of the systems’ behavior within the body and aids in assessing their suitability as potential drugs or in other applications related to absorption, distribution, metabolism, and elimination.

**FIGURE 2:**
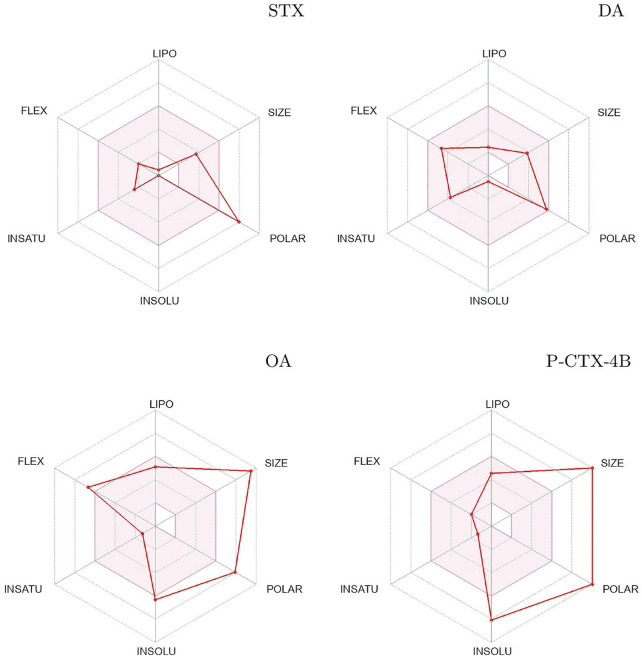
Bioavailability radars of Saxitoxin (STX), Domoic Acid (DA), Okadaic Acid (OA) and Ciguatoxin (P-CTX-4B).

The utilization of SwissADME prediction allows for the generation of a plot that serves as a measure of drug-likeness. This plot takes into account six essential physicochemical properties: lipophilicity, size, polarity, solubility, flexibility, and saturation. Each of these properties is assigned a specific range within the plot, collectively represented as the Bioavailability Radars (Figure 2). For a molecule to be considered drug-like, it must fall within the pink area encompassing all the defined ranges in the plot.

Upon analysis of the plots, it becomes apparent that all the studied systems exhibit a similar behavior, with minor variations observed in their solubility properties. However, it is noteworthy that molecule DA demonstrates an ideal arrangement within the plot, aligning perfectly with the desired ranges for each physicochemical property.

The assessment of lipophilicity, size, polarity, solubility, flexibility, and saturation is crucial in determining the drug-likeness of a molecule. These properties collectively influence factors such as absorption, distribution, and overall bioavailability. By examining how the studied systems align within the Bioavailability Radars plot, researchers can gain insights into their potential as viable drug candidates.

The Bioavailability Radar plots serve as a valuable tool for evaluating the drug-likeness of the studied systems based on their physicochemical properties. The ability of molecule DA to align favorably within the plot indicates its closer adherence to the desired ranges for each property, suggesting a higher likelihood of exhibiting optimal bioavailability and pharmacological activity.

In summary, the use of SwissADME prediction and the subsequent analysis of the Bioavailability Radar plots provide a comprehensive assessment of drug-likeness based on six physicochemical properties. While all the studied systems display similar behavior with minor differences in solubility, molecule DA stands out as the system that best aligns with the desired ranges for each property. This analysis aids in the evaluation and prioritization of molecules with higher potential for further development as drugs.

Finally, the prediction of the parameters related to the toxicity of the studied marine toxins has been achieved by resorting to the pkCSM software (Pires et al., 2015) and the results are displayed in Table 6:

**TABLE 6:** Toxicity parameters related to the marine toxins considered in this study.

Property	STX	DA	OA	P-CTX-4B	Units
AMES toxicity	Yes	No	No	No	Categorical
MTD (human)	0.190	0.394	-1.228	-1.732	log mg/kg/day
hERG I inhibitor	No	No	No	No	Categorical
hERG II inhibitor	Yes	No	No	Yes	Categorical
ORAT (LD50)	5.018	2.399	3.590	3.765	mol/kg
ORCT (LOAEL)	3.264	2.567	1.660	1.431	log mg/kg_bw/day
Hepatoxicity	No	Yes	No	No	Categorical
Skin Sensitisation	No	No	No	No	Categorical
*T. Pyriformis* toxicity	0.285	0.285	0.285	0.285	log μg/L
Minnow toxicity	5.406	2.516	2.967	7.586	log mM

AMES toxicity, often referred to as the Ames test, is a widely recognized and crucial assay in the field of toxicology. Named after its creator, Dr. Bruce Ames, this test assesses the mutagenic potential of chemicals or compounds. Mutagenicity is the ability of a substance to cause changes in an organism’s genetic material, typically DNA, which can lead to mutations and potentially contribute to the development of cancer. From Table 6, it can be appreciated that the only toxin displaying this behavior is STX, being negative for the other three molecules.

The Maximum Tolerated Dose (MTD) is a crucial concept within toxicity studies, particularly in the context of human safety assessment. It represents the highest dose of a substance that can be administered to humans in a clinical trial without causing unacceptable or severe adverse effects. Determining the MTD is a critical step in the development of pharmaceuticals and other compounds, as it helps establish the safety margin for human exposure. Toxicity studies in animals are typically conducted first to provide an initial assessment of potential risks. Based on these studies, researchers calculate the MTD by identifying the highest dose at which no life-threatening or severely debilitating side effects occur in animals. The MTD serves as a safety threshold, ensuring that human trials start at doses well below what could be harmful, and it plays a pivotal role in protecting the wellbeing of study participants. Additionally, it helps regulators make informed decisions about the approval and safe use of drugs and other products. From Table 6, we can conclude that DA excels from the other compounds.

In toxicity studies, hERG (human Ether-a-go-go-Related Gene) inhibition is a critical aspect of assessing the potential cardiac safety risks associated with new drugs and compounds. hERG refers to a specific ion channel in the heart, often denoted as hERG I (rapid delayed rectifier potassium current) and hERG II (the associated gene). These channels play a vital role in regulating the heart’s electrical activity. When a substance inhibits hERG I and II, it means that it interferes with the normal functioning of these channels. If a substance is found to have significant hERG inhibition, it raises concerns about its cardiac safety and the risk of inducing arrhythmias. Thus, the results from Table 6 indicates that none of the molecules studied here will be hERG I inhibitors, a different behavior occurs for hERG II where STX and P-CTX-4B will inhibit it and DA and OA will not.

Oral Rat Acute Toxicity (ORAT), often expressed as LD50 (Lethal Dose 50), is a key parameter in toxicity studies used to assess the potential harm of a substance when ingested by rats. The LD50 represents the dose of a compound required to cause the death of 50% of a group of test rats within a specified period, usually within 14 days. This metric is valuable for several reasons. Firstly, it helps researchers gauge the acute toxicity of a substance, providing a measure of how harmful it is when ingested. A lower LD50 indicates higher toxicity, while a higher LD50 suggests lower toxicity. Thus, DA can be considered as the drug with the highest toxicity according to this parameter.

Oral Rat Chronic Toxicity (ORCT) studies are vital in assessing the long-term health effects of substances when ingested over an extended period, typically several months to years. One of the key parameters measured in these studies is the LOAEL (Lowest Observed Adverse Effect Level). The LOAEL represents the lowest dose of a substance at which adverse effects or toxicological changes are observed in test rats during chronic exposure. These effects can include organ damage, alterations in biochemical parameters, or other health-related issues. The LOAEL serves as a critical reference point in evaluating the potential risks associated with chronic exposure to a particular substance. The highest value from Table 6 is for STX, and the lowest ones will be for OA and P-CTX-4B, with DA displaying an intermediate result.

Hepatoxicity is a significant concern in toxicity studies, as it refers to the potential for a substance to cause damage to the liver. The liver plays a crucial role in metabolizing and detoxifying many compounds, making it particularly vulnerable to toxic effects. Toxicity studies assess hepatoxicity by examining how a substance may affect the liver’s structure and function. This includes evaluating markers of liver damage such as elevated liver enzymes, histological changes, and alterations in biochemical parameters. When a substance is found to cause liver toxicity, it can lead to a range of adverse effects, including inflammation, fatty liver disease, fibrosis, or even more severe conditions like hepatitis or cirrhosis. It can be seen that DA present a positive value for this parameter reinforcing the observations mentioned earlier (Funk et al., 2014; Saeed et al., 2017; Grattan et al., 2018).

Skin sensitization is a vital aspect of toxicity studies, focusing on the potential of substances to induce allergic reactions when they come into contact with the skin. This allergic response is characterized by skin inflammation, itching, and redness, and it can be quite uncomfortable or even severe in some cases. Identifying skin sensitizers is crucial, as they can lead to contact dermatitis or other allergic skin reactions in individuals who are exposed to them. This is particularly relevant in industries such as cosmetics, chemicals, and pharmaceuticals, where products come into direct contact with the skin. In this case, none of the studied toxins will present skin sensitization.

Toxicity studies often include *T. pyriformis* as a test organism for assessing the potential ecological impact of various substances, particularly chemicals and pollutants. *T. pyriformis* is a species of ciliate protozoa commonly used in toxicity testing due to its sensitivity to environmental stressors and its role as an indicator organism. This information helps predict the potential impact of the substance on aquatic ecosystems, as *T. pyriformis* serves as a model organism for larger aquatic life forms. Thus, *T. pyriformis* toxicity studies are especially valuable in assessing waterborne pollutants and their effects on aquatic environments. From Table 6, the values are the same for the four molecules.

Minnow toxicity is a critical aspect of toxicity studies, particularly in the field of environmental science and aquatic ecology. These small, freshwater fish are often used as bioindicators to assess the health of aquatic ecosystems and the presence of contaminants. Minnow toxicity studies aim to understand how various substances, such as pollutants or chemicals, impact their survival, growth, and overall wellbeing. By examining Minnow toxicity, scientists can assess the ecological consequences of pollutants, identify sources of contamination, and develop strategies for mitigating their impact. These studies play a crucial role in safeguarding our freshwater resources and maintaining the delicate balance of aquatic ecosystems. It can be seen from the results on Table 6, that DA presents the lowest value in this test while P-CTX-4B displays the highest one.

## 4 Conclusion

This research comprehensively characterized four marine toxins, two hydrophilic and two lipophilic compounds, to investigate their drug-likeness and reactivity. Similarities in reactivity, drug-likeness, and pharmacokinetics persisted across diverse geometries. Domoic Acid (DA) had the lowest hardness, indicating strong target interaction and stability. DA had a low potential surface area, met Rule of Five parameters, and exhibited high gastrointestinal absorption (GI), with optimal properties in lipophilicity, molecular weight, polarity, solubility, saturation, and flexibility.

Computational tools enhance pharmacological research by reducing trial requirements, facilitating systematic exploration of drug candidate properties. In summary, this study sheds light on marine toxins’ drug-likeness and reactivity, emphasizing DA’s exceptional attributes. Computational tools are crucial for efficient drug development.

## Data Availability

The raw data supporting the conclusion of this article will be made available by the authors, without undue reservation.
